# Emotion Regulation through Movement: Unique Sets of Movement Characteristics are Associated with and Enhance Basic Emotions

**DOI:** 10.3389/fpsyg.2015.02030

**Published:** 2016-01-11

**Authors:** Tal Shafir, Rachelle P. Tsachor, Kathleen B. Welch

**Affiliations:** ^1^The Graduate School of Creative Arts Therapies, Faculty of Social Welfare and Health Sciences, University of HaifaHaifa, Israel; ^2^The Department of Psychiatry, University of MichiganAnn Arbor, MI, USA; ^3^Department of Theatre, School of Theatre and Music, University of Illinois at ChicagoChicago, IL, USA; ^4^Center for Statistical Consultation and Research, University of MichiganAnn Arbor, MI, USA

**Keywords:** emotion regulation, Laban Movement Analysis, motor characteristic, affect, movement, emotion, embodiment, bodily expression

## Abstract

We have recently demonstrated that motor execution, observation, and imagery of movements expressing certain emotions can enhance corresponding affective states and therefore could be used for emotion regulation. But which specific movement(s) should one use in order to enhance each emotion? This study aimed to identify, using Laban Movement Analysis (LMA), the Laban motor elements (motor characteristics) that characterize movements whose execution enhances each of the basic emotions: anger, fear, happiness, and sadness. LMA provides a system of symbols describing its motor elements, which gives a written instruction (motif) for the execution of a movement or movement-sequence over time. Six senior LMA experts analyzed a validated set of video clips showing whole body dynamic expressions of anger, fear, happiness and sadness, and identified the motor elements that were common to (appeared in) all clips expressing the same emotion. For each emotion, we created motifs of different combinations of the motor elements common to all clips of the same emotion. Eighty subjects from around the world read and moved those motifs, to identify the emotion evoked when moving each motif and to rate the intensity of the evoked emotion. All subjects together moved and rated 1241 motifs, which were produced from 29 different motor elements. Using logistic regression, we found a set of motor elements associated with each emotion which, when moved, predicted the feeling of that emotion. Each emotion was predicted by a unique set of motor elements and each motor element predicted only one emotion. Knowledge of which specific motor elements enhance specific emotions can enable emotional self-regulation through adding some desired motor qualities to one's personal everyday movements (rather than mimicking others' specific movements) and through decreasing motor behaviors which include elements that enhance negative emotions.

## Introduction

Following the ideas of Darwin (1872) and James (1884) that it is the afferent signals from the body which elicit emotions and feelings, several theorists in the field of emotion have postulated that sensory feedback from facial and postural movements contribute significantly to emotional experience (Tomkins, 1962; Laird, 1974; Izard, 1993). Indeed, a number of studies have demonstrated that feelings and attitude are affected by changing proprioceptive input from the muscles and joints through the adoption or mimicry of a certain facial expression, (McIntosh, 1996; Carr et al., 2003; Davis et al., 2009), posture (Riskind and Gotay, 1982; Duclos et al., 1989; Cacioppo et al., 1993; Stepper and Strack, 1993; Neumann and Strack, 2000; Duclos and Laird, 2001; Carney et al., 2010), head movement (Briñol and Petty, 2003; Forster, 2004), isometric muscle contraction in the arms (Cacioppo et al., 1993; Neumann and Strack, 2000), or certain expressive whole-body movements (Duclos and Laird, 2001; Shafir et al., 2013). It has further been demonstrated that combining both facial and bodily expressions of a certain emotion has a cumulative effect, producing stronger feelings of the corresponding emotion than do either bodily or facial expression alone (Flack et al., 1999). These studies have important clinical implication: such motor behavior can be easily used as a simple, readily available, free of adverse side-effects, inexpensive intervention for emotion regulation, i.e., for reducing inappropriate fear, anger, sadness and other negative emotions or increasing happiness, pride and other positive feelings (for a comprehensive review on emotion regulation through movement see Shafir, 2015).

While the effect of facial expression on feeling is already regularly used in Dialectical Behavioral Therapy (DBT), the therapeutic effects of motor execution of whole body expressions have been more difficult to implement: In facial expressions all people activate the same muscles to produce a certain facial expression (e.g., frowning, associated with anger, always involves the contraction of the corrugator muscle, and Duchenne smiling, associated with positive affect, is always achieved by contraction of the zygomatic major and the orbicularis oculi muscles). However, when it comes to whole body expressions, different people, or even the same person on different occasions and under different circumstances, may express the same emotion in a variety of movements and actions, using a variety of body parts. For example: anger can be expressed by hitting the table with a fist, slamming the door, stomping, forcefully kicking, etc. Indeed, the movements and postures that were used to elicit each emotion in the studies mentioned above were not always the exact same movements in each study.

This variety of motor choices available for elicitation or enhancement of each specific emotion raises two questions: (1) When it comes to the use of movement for emotion regulation—how do we know which specific bodily movements are associated with each emotion? (2) How can we determine and personalize the most effective and efficient movements for each individual to carry out in order to enhance or decrease each emotion? A possible solution to these questions would be to identify the motor characteristics common to movements that enhance a certain emotion, and to adopt these motor characteristics in our everyday movements when we aim to enhance the corresponding emotion, or to consciously avoid those motor qualities, when we aim to reduce the associated feeling. For example, rather than using sad-movements' qualities all day (e.g., slumped and closed posture) then setting aside time to practice mimicking a happy movement (e.g., jumping up while opening the arms), a person can consciously reduce or avoid the qualities of sad movements and adapt some qualities of happy movements (e.g., light rising posture) more often during regular daily activities such as walking, working on the computer or cleaning the house. In this study we aimed to identify motor characteristics common to movements whose execution enhances each of the four basic emotions.

While no study yet has tried to characterize movements whose motor execution *enhances* each emotion, there have been various attempts to characterize movements associated with the bodily *expression* of each emotion and/or its *perception* from body movements and posture. Some researchers, such as Darwin (1872), Wallbott (1998), or Dael (Dael et al., 2012a) identified several specific movements executed with specific body parts, which were associated with each individual emotion. Some used coding systems that included various movement dimensions, such as: movements in the vertical and sagittal direction, force, velocity, and directness (De Meijer, 1989), or: form, tempo, force, and direction (Montepare et al., 1999). A few used Laban movement analysis or its most known components: Effort and Shape (Levy and Duke, 2003; Gross et al., 2010, 2012; Crane and Gross, 2013). Yet others characterized the movements based on the specific muscles that are activated (Huis In 't Veld et al., 2014a,b), or used kinematic variables such as movement duration, velocity, acceleration, joints displacement (range of motion), and joint coordination (Pollick et al., 2001; Sawada et al., 2003; Roether et al., 2009; Gross et al., 2010, 2012; Barliya et al., 2013). Although these studies were able to discriminate among the different emotions expressed in movement, they used different coding schemes, making it difficult to compare outcomes across studies and to build a comprehensive description of the associations between certain motor characteristics of body movements and specific emotions (Gross et al., 2012). To overcome this difficulty, we characterized the movements in this study using Laban Movement Analysis (LMA), which, to the best of our knowledge, is the most comprehensive movement analysis system that exists (Bartenieff et al., 1984; Davis et al., 2007; Larboulette and Gibet, 2015).

Originally conceived by Rudolf Laban in the early to mid-twentieth century, and developed with Lisa Ullmann, Irmgard Bartenieff, Warren Lamb, F.C Lawrence and numerous others, LMA provides a well-established and widely accepted systematic language for describing and documenting movement (Bartenieff and Dori Lewis, 2002; Bradley, 2009; Studd and Cox, 2013; Fernandez, 2015). Analyzing movements using LMA is advantageous over other methods, as it captures various qualitative **motor elements** (movement characteristics) in addition to quantitative (kinematic) aspects of the movement. In addition, its vocabulary is descriptive and easily understood, making it suitable to be directly applied in movement coaching, research and therapy, and it is internationally recognized in various fields of studies including both the sciences and the arts. LMA categorizes movement descriptions into four main components: Body, Effort, Shape, and Space.

**Body** i.e., which ***body parts*** move, and **Space**, i.e., the directions in which the body moves through the general space in the sagittal (Forward/Back), vertical (Up/Down), and horizontal (Right side/Left side) planes, describe how the spatial-temporal body and limb relationships change in relation to one another and to the environment. The category of **Body** includes also specific common ***body actions*** such as Springing (jump, skip, etc.) Traveling (walking, running, crawling), Changing support (sitting, lying down, getting onto hands or knees), etc. **Effort** describes the qualitative aspect of movement, expressive of a person's inner attitude toward movement, and has four main factors, each describing the continuum between two extremes: fighting against the motor quality of that factor and indulging in that quality. The four components of Effort are: (1) activation of ***Effort-Weight***, or the amount of force or pressure exerted by the body, characterized as Strong, Light, or lack of weight activation, giving in to the pull of gravity i.e., Passive/Heavy; (2) ***Effort-Space***, describing the focus or attitude toward a chosen pathway, i.e., is the movement Direct or Indirect; (3) ***Effort-Time***, or the degree of urgency or acceleration/deceleration involved in a movement, i.e., is the movement Sudden or Sustained; and (4) ***Effort-Flow***, the element of control or the degree to which a movement is Bound, i.e., controlled by muscle contraction, vs. Free, i.e., being released/liberated. Finally, **Shape** refers to the way the body “sculpts” itself in space, i.e., it describes the changes in the relationship of the body parts to one another and to the surrounding environment that occur when a body moves (i.e., whether the body Encloses or Spreads, Rises or Sinks, Advances or Retreats).

LMA also examines additional movement patterns, such as the **phrasing** of the movement, which means the way movement elements are sequenced or change over time, analogous to phrasing in music (for a more detailed and systematic description of LMA see Bartenieff and Lewis, 1980; Studd and Cox, 2013; Fernandes, 2015). Thus, LMA is thorough, able to capture through its diverse motor elements all the movement assessments, dimensions and characteristics that were used in the previous studies described above. Indeed, in a recent study that used both Effort-Shape (part of LMA) and kinematic analyses to identify movement characteristics associated with positive and negative emotions experienced during walking, more differences among emotions were identified with Effort-Shape than kinematic analysis (Gross et al., 2012).

LMA's comprehensiveness as a motor analysis method could be inferred from its diverse use in research: it has been used to evaluate fighting behaviors of rats (Foroud and Pellis, 2003), to analyze behavior of non-human animals in naturalistic settings (Fagan et al., 1997), to diagnose autistic individuals (Dott, 1995), to evaluate motor recovery of stroke patients (Foroud and Whishaw, 2006), and to characterize the development of infants' reaching movements (Foroud and Whishaw, 2012). Several studies have also used it to describe, recognize or create bodily emotional expressions for applications in human-robot interactions, interactive games such as the Xbox, and in animations (Camurri et al., 2003; Zhao and Badler, 2005; Rett et al., 2008; Lourens et al., 2010; Zacharatos et al., 2013), and recently it has even been attempted, through the use of EEG, to identify the brain mechanisms underlying the production of some of the LMA motor elements (Cruz-Garza et al., 2014).

**Figure 1:**
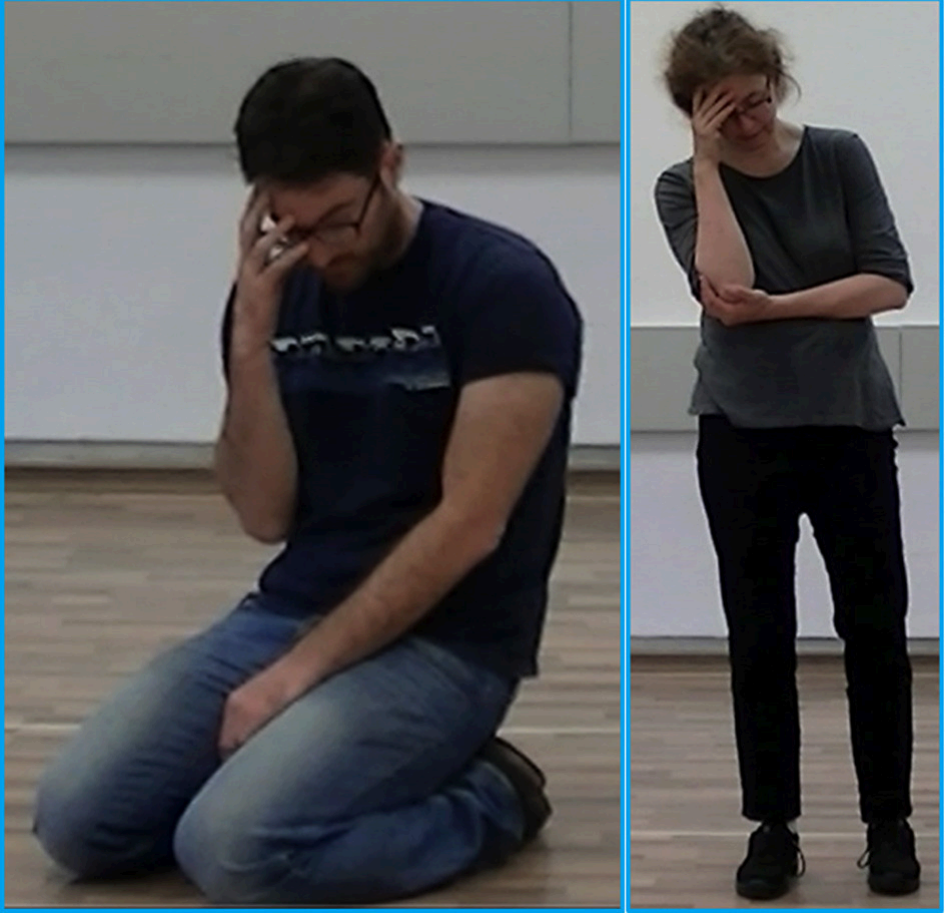
**This figure shows a picture of two different people doing two different movements, based on the same instruction—to move a movement that includes the motor elements: passive weight (lack of activation of weight effort), sinking (shape), head down (body part, space), and arm(s) to upper body (body action)**.

An additional advantage of LMA is its unique system for reading and writing movement, through the use of motifs. A motif is a written symbolic representation (using specific LMA symbols) that can be used as an instruction for the execution of a movement or a sequence of movements over time. Similar to the way a musical score describes how to play a sequence of notes over time, and may include both the notes to be played and the expressive quality intended by the composer, a motif may include the Body parts doing the movement and their actions, the change in the mover's Shape, the qualitative dynamics of the muscular contraction or Effort, and the advancement of the movement through Space. By asking people familiar with LMA to move specific motor elements (motor qualities) directly from a motif's symbols, rather than mimic a motor quality that they see in a video or learn from another person, their impression from each motor element is “uncontaminated” by any unintended influence of co-occurring other motor elements. In addition, by reading and moving motifs that include only motor qualities, participants can choose to move any movement they want, as long as the movement includes the required qualities. This enables researchers to base the study results on a variety of different movements, all having the same qualities, similar to the existing situation described above in which many different movements can elicit or enhance the same emotion. To illustrate this point Figure 1 shows pictures of two different people doing two different movements, based on the same instruction—to make a movement that includes the motor elements: Passive weight (effort), Sinking (shape), Head down (body part and Space), and Arm(s) to upper body (body action).

Thus, this study aimed to identify Laban motor elements (i.e., the motor characteristics or motor attributes) characterizing movements whose execution enhances each emotion. To this end, we chose to characterize movements that enhance four basic emotions: anger, fear, happiness, and sadness. To achieve this goal, we asked people familiar with LMA to move specific motor elements, and to answer which emotion they felt while moving each movement and the intensity of the emotion they felt.

## Materials and methods

### Participants

Overall, 80 people participated in the study. The first group of volunteers had 27 participants (24 females, 3 males; 17 US residents, 10 non-US residents; age range 26–60 years old), who answered the questionnaires during latter stages of their training to become Certified Movement Analysts (CMA) at the Laban/Bartenieff Institute of Movement Studies, located in New York. These participants had prior experience of at least 220 h of training in reading and performing movement annotations (motifs). Reading and performing movements from motifs is similar to reading and performing music from a score. In order to analyze the high number of variables involved in the study of complex movement (like the ones analyzed in the present study), we needed a higher number of subjects than were available through the Laban/Bartenieff Institute of Movement Studies. Thus, we recruited additional 53 participants (52 females, 1 male, 1 unidentified; 35 US residents, 17 non-US residents, 1 unidentified; age range 25–86 years old) via an on-line survey, disseminated through list-serves and websites for movement professionals. Participants in the survey rated their own level of experience with LMA as a beginner, intermediate or advanced. Overall, the study included 10 beginner participants, 41 intermediate, and 29 advanced Laban movement analysts, from all over the world (Europe, Australia, North America and South America).

All participants consented to take part in the study, which was approved by the University of Michigan Institutional Review Board.

### Experimental design and procedures

The identification of motor elements characterizing the expression of each emotion: anger, fear, happiness, and sadness were done in two phases. In the first phase, motifs were created for each emotion: motifs of single motor elements potentially meaningful for the motor expression of that emotion and motifs of various combinations of those elements were both created. In the second phase, these motifs were read and moved by people who know how to read motif notation. After reading and moving the motifs, participants rated the emotion that the movement elicited in them and the intensity of that emotion.

During the first phase, to create motifs characterizing the basic emotions, six expert CMAs, who did not participate in the second phase of the study, viewed a validated set of 3-s video clips of actors expressing emotions through whole-body movements. In these clips, validated by Atkinson et al. (2007), all movements were presented on a black background, and were performed by male and female actors who wore uniform dark gray, tight-fitting clothes and headwear, so that facial expressions were not visible. Ten video clips of each emotion: anger, fear, happiness and sadness were viewed by the experts, to identify possible meaningful motor elements that could have contributed to the perception of the specific emotion. The six experts decided which are the potentially meaningful elements based on two criteria: First, they identified the motor elements that were common to (appeared in) all clips expressing the same emotion. Second, they analyzed and annotated the movements of the two best-recognized clips (as rated by Atkinson's subjects during the validation process) for each emotion: one clip performed by a male actor and one performed by a female actor.

**Figure 2:**
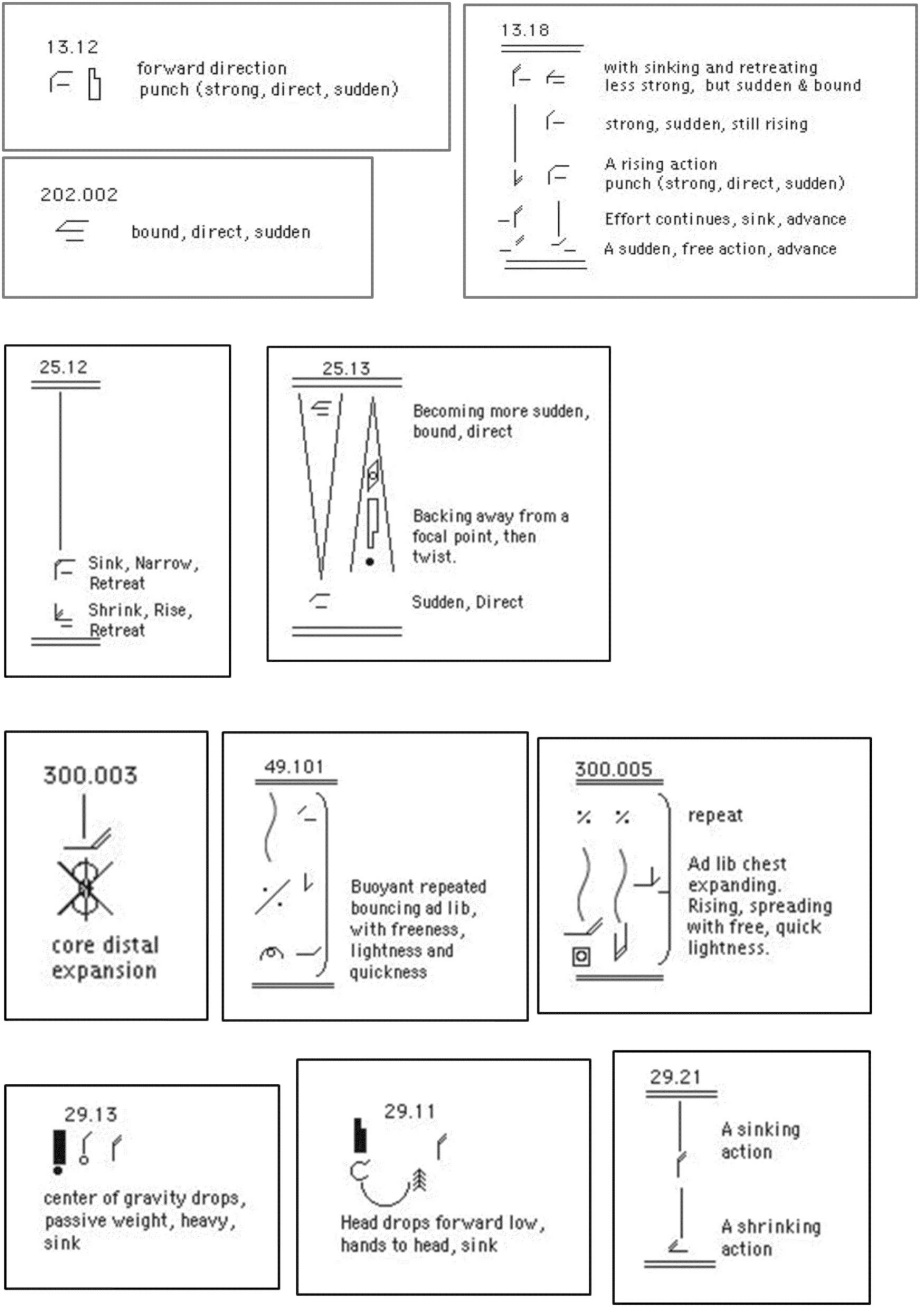
**Examples of motifs that were read and moved by the participants**. The first row of “angry” motifs (# 13.12, 202.002, and 13.18) are motifs constructed from motor elements that were taken from angry clips. The second row of “fearful” motifs (# 25.12 and 25.13) are motifs constructed from motor elements that were extracted from fearful clips. The third row of “happy” motifs (# 300.003, 49.101, and 300.05) are motifs constructed from motor elements that were extracted from happy clips. The last row of “sad” motifs (# 29.13, 29.11, and 29.21) are motifs constructed from motor elements that were extracted from the sad clips. Reading the motifs and moving them is similar to reading musical score and singing or playing the notes in it. Motifs that included 1–3 motor elements (e.g., motifs 13.12, 202.002, 300.003, 29.13, 29.11, 29.21) could be moved by the participants using a variety of movements, all having the same qualities. For example, moving the qualities of punch (strong, direct, sudden) and forward direction (motif 13.12) could be done by punching a fist forward with one arm, punching with two arms together or one arm after another, a sharp Karate like strike forward with the edge of the hand, a fast kick forward, etc. Moving the elements head drop, bringing hands to the head and sinking (motif 29.11) could yield any of the two postures shown in Figure 1. Participants could repeat the same movement again and again or could move a variety of movements one after another but all having the same motor elements. Reading and moving the more complex motifs (e.g., motifs 13.18, 25.12, 25.13, 300.005) which include instructions (read from the bottom up) for a sequence of movements, can be paralleled to playing from a musical score a whole musical phrase.

In order to determine which specific motor elements and/or combinations of elements are crucial to the experience of each emotion, motifs showing the motor elements described above were created with different combinations of elements in them. Some of the motifs that were created for this study were very basic and included only one motor element or quality of movement (e.g., light effort; condensing the body; or retreating in space), some included a combination of motor qualities (e.g., retreating in space while condensing the body at the same time), and some more complex motifs included a short sequence of movements, taken from the most recognized clips, and the description of the qualitative aspects of their performance. By the end of this phase we had nine motifs created from motor elements that express anger (“angry” motifs), eight “fearful” motifs, 13 “happy” motifs and 10 “sad” motifs. For examples of these motifs see Figure 2.

During the second phase of the study participants read these motifs, moved them, and answered a forced-choice questionnaire in which they rated which emotion they felt while moving the motif, and the intensity of the emotion they felt on a scale of 1–5. To facilitate the reading of motifs, for each motor element in the motif we added to the side of the symbol a verbal description of that element (Figure 2). Reading the motifs and moving them is similar to reading musical score and singing or playing it. Participants were asked to move the motifs repeatedly until the movement elicited a certain emotion. Motifs that included 1–3 motor qualities (e.g., motifs 13.12, 202.002, 300.003, 29.13, 29.11, 29.21, in Figure 2) could be moved by the participants using a variety of movements, all having the same qualities. For example, moving the qualities of punch (strong, direct, sudden) and forward direction (motif 13.12 in Figure 2) could be done by punching a fist forward with one arm, punching with two arms together or one arm after another, a sharp Karate like strike forward with the edge of the hand, a fast kick forward, etc. Moving the elements head drop, bringing hands to the head and sinking (motif 29.11 in Figure 2) could result in postures similar to the two shown in Figure 1. Participants could repeat the same movement again and again or could move a variety of movements one after another but all having the same motor elements. Moving the more complex motifs (e.g., motifs 13.18, 25.12, 25.13, in Figure 2) which include instructions (read from the bottom up) for a sequence of movements, can be paralleled to playing from a musical score a whole musical phrase. Participants were given as much time as they needed to read each motif, repeatedly move it, and answer the questions concerning the emotion felt in response to the movement and its intensity. Because the process of reading and moving a motif until a clear emotion is felt varied among participants and could have taken up to a few minutes (depending on the person's personality and level of experience with Laban Movement Analysis, and depending on the motif's complexity), and because we had many (40) different motifs, we could not have expected each participant to read, move and rate all motifs (participants participated in the study on a pure voluntary basis, with no compensation for their time). Assuming that each participant would complete only a few motifs and to ensure enough readings of each motif, we randomized the order of motifs in the survey, so that each participant got different motifs in the beginning of the survey, and therefore read and moved different motifs.

### Data reduction and analysis

Each motif was coded for the motor elements that appeared in it. Overall 29 motor elements were coded (Table 1): nine Effort elements—two Effort-flow: bound and free; two Effort-weight: strong, light, and the lack of weight activation: passive weight/heavy; two Effort-time: sudden and sustained; and two Effort-space: direct and indirect. Eight Shape elements—two Shape-change: expand and condense; two Shape-vertical: rise and sink; two Shape-horizontal: spread and enclose; and two Shape-sagittal: advance and retreat. Five Space elements—two Space-vertical: up and down; two Space-sagittal: forward and backward, and one Space-rotation: twist. Five Body elements—three Body-parts: core, arms and head; and two Body-actions: arm(s) to upper body, and jump. Two Phrasing elements—one Phrasing-intensity: increasing; and one Phrasing-rhythmicity: reinitiating.

**Table 1:** **Motor elements used in the study**.

Effort	Flow	Bound
		Free
	Weight	Strong
		Light
		Passive/Heavy
	Time	Sudden/Quick
		Sustained
	Space	Direct
		Indirect
Shape	Shape Change	Expand
		Condense
	Vertical	Rise
		Sink
	Horizontal	Spread
		Enclose
	Sagittal	Advance
		Retreat
Space	Vertical	Up
		Down
	Horizontal	Side open
		Side across
	Sagittal	Forward
		Back
	Rotation	Twist
Body	Body parts	Core
		Arm(s)
		Head
	Body action	Arm to upper body
		Jump
Phrasing	Intensity	Increase
		Decrease
	Rhythmicity	Reinitiating

*The left column in the table includes the main components of LMA. Each component includes several factors/categories of motor qualities which are described in the middle column of the table. Each factor describes the continuum between two extremes. In Effort, one extreme fights against the motor quality of that factor and the other extreme indulges in that quality. The right column in the table depicts the specific motor elements that constitute those extremes. For example, one of the motor qualities which describe the Effort that is invested in the movement is the Flow of the movement. When the movement is Bound there is a feeling of fighting against the flow of the movement; when the movement is Free, it creates a flow in the movement*.

Since some of the motifs were based on the original video clips that lasted three seconds, the duration of each motif was divided into three segments for coding. For each segment, we coded the motor elements that were present in it: Motor elements that appeared in a segment of the motif were coded as 1 for that segment, and motor elements that didn't appear were coded as 0. Thus, every motif had a total score for each motor element quantifying the prevalence of that motor element in the motif. This score could have had the value of 0 if the motor element didn't appear in the motif at all, the value of 1 if it appeared in only one third of the duration of the movement, the value of 2 if it appeared during two thirds of duration of the movement annotated in the motif, and the value of 3 if the motor element appeared along the entire duration of the movement. Motifs that included only a certain motor quality, without specifying how the movement changes over time (horizontal motifs) were coded as if each element that appeared in them lasted throughout the entire motif duration, i.e., elements that appeared in them got the score of 3. Based on this coding system, each motif that was read, moved and emotionally rated by a certain participant had a score for all 29 motor elements mentioned above, where each score got a value between 0 and 3.

To determine which motor elements contribute to the enhancement of each emotion, a logistic regression model was fitted to predict each emotion (anger, fear, happiness, and sadness) felt during the movement of a certain motif, using each motor element score (e.g., the score for “Effort-flow: free”) as a predictor. Because each participant performed multiple motifs, we used a GEE (Generalized Estimating Equations) model to take into account correlations among the responses for each subject. To adjust for the multiple tests for each emotion (29 motor elements as predictors), we applied the Bonferroni correction to the *p*-values, and therefore *p*-value of 0.0017 (0.05/29 = 0.0017) or less was considered to be statistically significant. All analyses were run using SAS 9.2 for Windows. (SAS Institute, Inc., Cary, North Carolina.)

## Results

Altogether, we had 1241 reads of the motifs: 290 reads of “angry” motifs, (i.e., motifs that were composed of motor elements extracted from angry movements as identified and validated by Atkinson et al., 2007), 251 reads of “fearful” motifs, 396 reads of “happy” motifs, and 304 reads of “sad” motifs. Different motifs were rated by different numbers of subjects, but on average, each motif was moved and rated by 25 subjects. Based on these ratings, 20 out of the 29 motor elements were found to be significant predictors for feeling a specific emotion as a result of moving the motifs (Table 2). Each emotion was predicted by a unique set of motor elements, and each motor element was a significant predictor for only one emotion: none of these motor elements significantly predicted more than one emotion. Seven out of the nine “Effort” motor elements, six out of eight “Shape” motor elements, two out of five “Space” motor elements, three out of five “Body” elements, and one out of two Phrasing elements were found to be significant predictors.

**Table 2:** **Motor elements that predict each emotion**.

**Emotion**	**Motor element**	**Estimate**	**Stderr**	**Lower CL**	**Upper CL**	***Z***	***P*-value**
Anger	Effort-Weight:Strong	1.1388	0.1091	0.9249	1.3527	10.43	<0.0001
Anger	Effort-Time:Sudden	0.8119	0.1648	0.4889	1.1349	4.93	<0.0001
Anger	Shape-Sagital:Advance	0.7609	0.2033	0.3624	1.1594	3.74	0.0002
Anger	Effort-Space:Direct	0.7511	0.1714	0.4152	1.0870	4.38	<0.0001
Fear	Shape-Sagital:Retreat	0.8506	0.1013	0.6521	1.0492	8.40	<0.0001
Fear	Shape-Change:Condense	0.7775	0.1650	0.4541	1.1008	4.71	<0.0001
Fear	Effort-Flow:Bind	0.7691	0.1913	0.3942	1.1440	4.02	<0.0001
Fear	Space-Sagital:Back	0.7355	0.1563	0.4292	1.0417	4.71	<0.0001
Fear	Shape-Horizontal:Enclose	0.6673	0.1818	0.3110	1.0235	3.67	0.0002
Happiness	Body-Action:Jump	1.5676	0.3901	0.8029	2.3322	4.02	<0.0001
Happiness	Phrase-Rhythmicity:Reinitiating	1.0527	0.2275	0.6068	1.4985	4.63	<0.0001
Happiness	Shape-Horizontal:Spread	0.9202	0.1967	0.5348	1.3057	4.68	<0.0001
Happiness	Effort-Flow:Free	0.9031	0.2024	0.5063	1.2998	4.46	<0.0001
Happiness	Effort-Weight:Light	0.8489	0.2049	0.4474	1.2504	4.14	<0.0001
Happiness	Space-Vertical:Up	0.7313	0.2073	0.3250	1.1376	3.53	0.0004
Happiness	Shape-Vertical:Rise	0.6943	0.2171	0.2688	1.1198	3.20	0.0014
Sadness	Effort-Weight:Passive	1.0635	0.1566	0.7566	1.3703	6.79	<0.0001
Sadness	Body-Action:Arms to upper body	1.0045	0.1853	0.6414	1.3676	5.42	<0.0001
Sadness	Shape-Vertical:Sink	0.9287	0.1837	0.5687	1.2888	5.06	<0.0001
Sadness	Body-Parts:Head	0.7372	0.1888	0.3672	1.1073	3.91	<0.0001

*This table describes the motor elements that significantly predicted each emotion based on the logistic regression that was fitted to predict the emotion (anger, fear, happiness, or sadness) felt during the movement of a certain motif. The regression was fitted using each motor element score (e.g., the score for “Effort-flow: free”) as a predictor. As can be seen from the table, each emotion was predicted by a unique set of motor elements, and each motor element was a significant predictor for only one emotion: none of the motor elements significantly predicted more than one emotion. Stderr, Standard error of the estimate; LowerCL, lower confidence interval; UpperCL, upper confidence interval*.

*Anger* was significantly predicted by four motor elements. Three Effort elements: Strong (*B* = 1.139, *Z* = 10.43, *p* < 0.0001), Sudden (*B* = 0.812, *Z* = 4.93, *p* < 0.0001), and Direct (*B* = 0.751, *Z* = 4.38, *p* < 0.0001), and one Shape element: Advance (*B* = 0.761, *Z* = 3.74, *p* = 0.0002).

*Fear* was significantly predicted by five elements: one Effort element: Bind (*B* = 0.769, *Z* = 4.02, *p* < 0.0001), three Shape elements: Retreat (*B* = 0.851, *Z* = 8.40, *p* < 0.0001), Condense (*B* = 0.778, *Z* = 4.71, *p* < 0.0001), and Enclose (*B* = 0.667, Z = 3.67, *p* = 0.0002), and one Space element: Back (*B* = 0.736, *Z* = 4.71, *p* < 0.0001).

*Happiness* was significantly predicted by seven elements: two Effort elements: Free (*B* = 0.903, *Z* = 4.46, *p* < 0.0001) and Light (*B* = 0.849, *Z* = 4.14, *p* < 0.0001), two Shape elements: Spread (*B* = 0.920, *Z* = 4.68, *p* < 0.0001), and Rise (*B* = 0.694, *Z* = 3.20, *p* = 0.0014), one Space element: Up (*B* = 0.731, *Z* = 3.53, *p* = 0.0004), one Body element: Jump (*B* = 1.568, *Z* = 4.02, *p* < 0.0001), and one Phrasing characteristic: Rhythmicity/Reinitiating (*B* = 1.053, *Z* = 4.63, *p* < 0.0001).

*Sadness* was also significantly predicted by four motor elements. Effort of Passive weight rather than activated weight (*B* = 1.064, *Z* = 6.79, *p* < 0.0001), one Shape element: Sink *B* = 0.929, *Z* = 5.06, *p* < 0.0001), and two Body elements: Arm(s) to upper body (*B* = 1.005, *Z* = 5.42, *p* < 0.0001), and Head (down) (*B* = 0.737, *Z* = 3.91, *p* < 0.0001).

Table 3 depicts the motor elements which were found as significant negative predictors, i.e., when they appear in a movement, the given emotion is significantly **Less** likely to be felt. In other words, these motor elements predict NOT feeling the particular emotion. This table includes both motor elements that served as positive predictors for other emotions, as well as motor elements that didn't positively predict any emotions. In addition, while each motor element in Table 2 was a significant positive predictor for only one emotion, in Table 3 some of the motor elements serve as negative predictors for two or even three emotions.

**Table 3:** **Motor elements that were significant negative predictors for each emotion**.

**Motor element**	**Estim anger**	***p*-value Anger**	**Estim fear**	***p*-value Fear**	**Estim happy**	***p*-value happy**	**Estim sad**	***p*-value sad**
Effort-Time:Sustained	−1.7576	<0.0001						
Body-Action:Jump	−1.3312	<0.0001					−1.6859	<0.0001
Phrase-Rhythmicity:Reinitiating	−1.2411	<0.0001	−1.2435	0.0001			−1.1089	0.0011
Body-Parts:Core	−0.9385	0.0002						
Effort-Weight:Light	−0.9294	<0.0001						
Shape-Vertical:Sink	−0.9266	0.0008						
Shape-Horizontal:Spread	−0.9011	0.0002					−0.9164	0.0008
Shape-Horizontal:Enclose	−0.8381	<0.0001						
Body-Parts:Head	−0.8127	<0.0001						
Body-Action:Arms to upper body	−0.7896	<0.0001			−2.1644	<0.0001		
Effort-Weight:Passive	−0.7765	0.0002	−1.1694	0.0001	−1.4368	0.0009		
Shape-Sagital:Retreat	−0.4993	0.0012						
Effort-Flow:Bind					−1.3583	<0.0001		
Effort-Space:Direct					−1.0154	0.0001		
Effort-Weight:Strong					−0.5420	0.0003		
Shape-Sagital:Advance							−2.9305	0.0006
Phrase-Intensity:Increase							−1.3483	<0.0001
Effort-Time:Sudden							−1.2907	<0.0001
Space-Vertical:Up							−0.9638	0.0014
Shape-Change:Expand							−0.8550	0.0013

*This table describes the motor elements that were significant negative predictors of each emotion, i.e., when they appear in a movement, the given emotion is significantly ***Less*** likely to be felt. For each motor element in the left column, its estimate (Estim) and significance value (p-value) are shown when that motor element was a significant negative predictor for a certain emotion. When the motor element was not a significant negative predictor for an emotion the associated cell in the column of that emotion was left empty. In contrast to the positive predictors described in Table 2, where each motor element was a significant predictor for only one emotion, some of the motor elements serve as significant negative predictors for two or even three emotions*.

As a preliminary exploration, to probe how many elements and which combinations of elements need appear in a movement sequence in order for that movement sequence to enhance an emotion, we created a descriptive table showing each motif in the study, with the motor elements appearing in that motif and the quantified score of their appearance (as described in the Material and Methods section). Since the motor elements of each motif were originally determined from their appearance in movements expressing a specific emotion in one of Atkinson's validated video-clips, we also calculated the percentage of subjects who “correctly” identified the emotion by moving each motif, i.e., the percentage of subjects who felt the same emotion as the emotion expressed by the actors' movements from which those motor elements were extracted (% correct). For each motif, we multiplied the percent of subjects that “correctly” identified (felt) the emotion expressed in the motif by the average emotional intensity as was rated by those subjects who “correctly” felt the emotion. The result of this multiplication was used as a measure for the strength of the association between each motif and its specific associated emotion. In other words, the higher the result of this “%correct^*^intensity_felt” multiplication, the higher the chance that people who move the motif will strongly feel the associated emotion. We then ordered the motifs composed of motor elements that were extracted from movements expressing a certain emotion, based on this measure.

Table 4 displays the four motifs for each emotion that got the highest result for this “%correct^*^intensity_felt” measure. For each motif in this table we show all the motor elements included in it, which scored more than 0 (i.e., that appeared in the motif at least once). Each motor element in the table is accompanied with its score for that motif. The motor elements that were found as significant predictors for that emotion are presented on a yellow background. We decided to use 80% recognition and the median rated intensity, 3, to calculate a threshold number of 2.4 (0.8 ^*^ 3 = 2.4), as the threshold for defining which motifs brought about the experience of the target emotion. As can be seen, the “%correct^*^intensity_felt” measure for all the motifs presented in Table 4 were equal to or bigger than the 2.4 threshold, i.e., moving these motifs brought about the feelings of their associated emotion. Further implications for the data presented in this descriptive table will be discussed in the Discussion section.

**Table 4:** Motifs having the highest “%correct^*^intensity_felt” for each emotion.

Anger	Motif number	13.012	202.001	13.003	200.001
	Number of cases	31	28	33	24
	%correct^*^intensity felt	2.73	2.53	2.42	2.40
		Effort-Weight:Strong-3	Effort-Weight:Strong-3	Effort-Weight:Strong-3	Effort-Weight:Strong-3
		Effort-Time:Sudden-3	Effort-Time:Sudden-3	Effort-Time:Sudden-3	Effort-Time:Sudden-3
		Effort-Space:Direct-3	Effort-Flow:Bind-3	Effort-Space:Direct-3	Effort-Space:Direct-3
		Space-Sagital:Forward-3			Shape-Sagital:Advance-3
					Space-Sagital:Forward-3
					Shape-Change:Expand-3
					Body-Parts:Arm-3
					Phr-Intensity:Increase-2
Fear	Motif number	48.007	48.102	48.001	48.002
	Number of cases	30	30	30	30
	%correct^*^intensity felt	2.93	2.78	2.70	2.58
		Shape-Change:Condense-3	Shape-Change:Condense-3	Effort-Flow:Bind-3	Space-Sagital:Back-3
		Shape-Sagital:Retreat-3	Sjape-Sagital:Retreat-3	Shape-Change:Condense-3	Effort-Flow:Bind-2
		Effort-Flow:Bind-2	Space-Sagital:Back-3	Shape-Horizontal:Enclose-3	Effort-Time:Sudden-3
		Effort-Time:Sudden-3	Effort-Flow:Bind-2	Shape-Sagital:Retreat-3	Body-parts:Arm-3
		Body-parts:Arm-3	Effort-Time:Sudden-3	Space-Sagital:Back-2	Phr-Intensity:Increase-2
		Effort-Space:Direct-2	Phr-Intensity:Increase-2	Body-Action:ArmtoUpBd-3	Effort-Space:Direct-2
		Phr-Intensity:Increase-2	Space-Rotation:Twist-2	Effort-Space:Direct-3	Space-Rotation:Twist-2
			Effort-Space:Direct-2	Shape-Vertical:Sink-3	
			Body-parts:Arm-1	Effort-Time:Sudden-2	
				Body-parts:Arm-2	
				Body-parts:Head-2	
				Space-Rotation:Twist-2	
				Body-Parts:Core-1	
				Effort-Time:Sustained-1	
				Space-Sagital:Forward-1	
Happiness	Motif number	302.001	27.026	27.025	49.101
	Number of cases	8	29	31	34
	%correct^*^intensity felt	3.88	3.03	3	2.97
		Body-Action:Jump-2	Effort-Weight:Light-3	Effort-Weight:Light-3	Space-Vertical:Up-3
		Shape-Change:Expand-2	Body-Action:Jump-1	Shape-Vertical:Rise-2	Phr-Rhyth:Reinitiating-3
		Effort-Time:Sudden-1	Phr-Rhyth:Reinitiating-1	Shape-Horizontal:Spread-2	Body-Action:Jump-2
		Shape-Sagital:Advance-1	Effort-Flow:Free-1	Body-Action:Jump-1	Effort-Weight:Light-1
		Body-Parts:Core-1	Effort-Time:Sustained-1	Phr-Rhyth:Reinitiating-1	Effort-Flow:Free-1
		Body-Parts:Arm-1	Space-Vertical:Down-1	Effort-Flow:Free-1	Effort-Time:Sudden-1
			Space-Rotation:Twist-1	Shape-Change:Expand-2	Space-Rotation:Twist-1
				Effort-Time:Sustained-1	
				Shape-Sagital:Retreat-1	
				Space-Vertical:Down-1	
				Space-Rotation:Twist-1	
Sadness	Motif number	29.013	29.001	29.017	29.022
	Number of cases	38	32	26	26
	%correct^*^intensity felt	3.05	2.96	2.92	2.76
		Effort-Weight:Passive-3	Effort-Weight:Passive-3	Body-Action:ArmtoUpBd-3	Body-Parts:Head-3
		Shape-Vertical:Sink-3	Shape-Vertical:Sink-3	Body-Parts:Head-3	Body-Action:ArmtoUpBd-3
		Space-Vertical:Down-3	Body-Parts:Head-3	Shape-Vertical:Sink-1	Effort-Weight:Passive-1
		Body-Parts:Core-3	Body-Action:ArmtoUpBd-3	Body-Parts:Arm-3	Shape-Vertical:Sink-1
			Body-Parts:Arm-3	Space-Vertical:Down-1	Body-Parts:Arm-3
				Shape-Change:Condense-1	Space-Vertical:Down-1
					Effort-Flow:Free-1
					Shape-Change:Condense-1

For each motif in this table we show how many people moved this motif (number of cases), its “%correct^*^intensity_felt” score, and the motor elements that were included in it which scored more than 0, i.e., that appeared in the motif at least once. Each motor element in the table is accompanied with its score for that motif. The motor elements that were found as significant predictors for that emotion are presented on a yellow background. The “%correct^*^intensity_felt” score represents the multiplication of the percent of subjects that “correctly” identified (felt) the emotion expressed in the motif by the average emotional intensity as was rated by those subjects who “correctly” felt the emotion. The result of this multiplication was used as a measure for the strength of the association between each motif and its specific associated emotion. The table displays the four motifs for each emotion that got the highest result for this “%correct^*^intensity_felt” measure. A threshold of 2.4 was calculated for this measure to define which motifs brought about the experience of the target emotion (for more details see the last paragraph of the Result section). As can be seen, the “%correct^*^intensity_felt” measure for all the motifs presented in this table were equal to or bigger than the 2.4 threshold, i.e., moving these motifs brought about the feelings of their associated emotion. Implications for the data presented in this descriptive table are reviewed in the Discussion section.

## Discussion

In this study we aimed to identify Laban motor elements characterizing movements whose execution enhances each of the emotions: anger, fear, happiness, and sadness. To this end, we asked people familiar with LMA to read and move motifs that included different combinations of motor elements and to rate what emotion was evoked in them while moving each motif, and the intensity of the evoked emotion. Based on statistical analysis of this data we found a set of motor elements associated with each emotion which, when moved, predict the feeling of that emotion.

As can be seen in Table 2, each emotion was predicted by a unique set of motor elements, and each motor element was a significant predictor for only one emotion. Moreover, as can be seen in Table 3, many of the motor elements that positively predicted a certain emotion served as significant negative predictors for one or more of the other emotions. This distinctive one-to-one matching between each emotion and certain motor characteristics, i.e., certain type of proprioceptive feedback, supports the idea that the four basic emotions investigated in this study are discrete and biologically based.

Because many of the recent papers that have used Laban Movement Analysis (LMA) concentrated on or emphasized predominantly the analysis of the Effort category (Zhao and Badler, 2005; Barakova and Lourens, 2010; Lourens et al., 2010; Crane and Gross, 2013; Zacharatos et al., 2013; Cruz-Garza et al., 2014), it is important to note that the predictors we found are from all categories of LMA: Body, Effort, Space and Shape. We found seven Effort, seven Shape, two Space, three Body and one Phrasing predictors. Another important finding is that no emotion was predicted by two opposite motor elements. Opposite qualities of movement always distinguished between the emotions: While rising, and light movements predicted happiness, sinking and heavy (passive weight) movements predicted sadness. While advancing movements predicted anger, retreating predicted fear. As opposed to fear, which was predicted by condensing and enclosing in bound flow, happiness was predicted by spreading in free flow, and although both happiness and anger are considered to be “approach emotions,” their expression in movement could be distinguished based on the Effort-weight quality: angry movements were strong while happy movements were light. Similarly, although both sadness and fear are considered to be “avoidance emotions,” their expression in movement could also be distinguished based on the Effort-weight quality, or rather its absence: passive weight was a positive predictor for sadness but a negative predictor for fear. Another finding that deserves attention was that rhythmicity was not only a positive predictor for happiness, but also a negative predictor for all other emotions. This finding indicates a very strong and unique association between rhythmicity and happiness. Similar unique and strong association was found between passive/heavy weight and sadness, as this motor element positively predicted sadness and negatively predicted all other emotions.

The specific motor elements that were found to evoke or enhance each emotion are not surprising, and match what we already know intuitively. These elements characterize motor behaviors that, based on evolutionary theories, are associated with the specific emotions. Moreover, these elements describe movements that have been found in previous studies to elicit the specific emotions, as well as movements that have been identified as expressions of those emotions. Yet, this is the first study to demonstrate scientifically that it is the motor qualities of any movement, and not some specific fixed movements, that can evoke or enhance specific emotion and feelings.

Feeling angry was predicted by *advancing* with a *strong sudden* and *direct* effort. Anger is known as an approach (advancing) emotion, and a punching movement, which is a frequent universal expression of anger, is characterized by a strong, sudden and direct effort. The combination of these three specific efforts is also known in LMA as an “action drive,” and supposedly characterizes purposeful movements and actions that are driven by a certain aim. Indeed, according to some theories the purpose of anger is to drive us to action: to fight for survival, or to act aggressively toward others, in order to cause them to behave in a way that will resolve conflicts of interest in favor of the angry individual (Sell et al., 2009).

Punching movements and leaning forward (which is basically advancing in the shape of the body) were used in previous studies to elicit anger (Duclos et al., 1989; Flack et al., 1999; Duclos and Laird, 2001), but in those studies they were described as specific movements and not by the motor qualities that characterize those movements. Previous studies that described anger expressions portrayed angry movements as *strong, fast*, and *direct* movements (De Meijer, 1989; Crane and Gross, 2013), as consisting of shaking the fists and stamping the feet (Atkinson et al., 2004) which are also *strong, sudden, and direct* movements, and as including leaning forward (Winters, 2008), bending the head forward (Kleinsmith et al., 2006; Roether et al., 2009), or stretching the arms forward (De Meijer, 1989; Wallbott, 1998; Atkinson et al., 2004; Gross et al., 2010), which can all be described as movements during which the shape of the body *advances*.

Feeling fear was predicted by *enclosing* and *condensing* the body, as well as by moving *backward* in space and *retreating* in the shape of the body (i.e., leaning backwards with the torso). All of these motor characteristics describe well-known responses to danger in the animal kingdom. By enclosing and condensing its body the frightened animal tries to make itself as small, non-visible, and unthreatening as possible, in order to avoid a battle with a stronger animal. Moving backwards in space with the entire body, or shaping the torso backward, are also done in order to avoid conflict, or avoid being hurt especially in the vital organs. Feeling fear was also predicted by *bound flow*, a motor quality that is achieved by intense muscle activation which is also necessary for freezing—another typical response to a dangerous situation in the animal kingdom. Thus, all the motor elements that predicted feeling fear describe motor responses to a dangerous situation, and similar to the increased activation of the sympathetic nervous system in response to danger and its interoception in the brain, the proprioceptive feedback from such movements, based on James's theory (James, 1884), should also evoke the feeling of fear.

Again, these motor qualities describe also the movements used in previous studies for fear elicitation. Both Duclos and Flack asked their subjects to lean backward (*retreat*) and dip the shoulders (*condense*) in order to create the posture that elicited fear (Duclos et al., 1989; Flack et al., 1999). Similarly, these motor qualities describe also the movements which have been found in previous studies to characterize fear expressions: Both Atkinson and De Meijer described the fear movements as involving moving *backward* in contracted or closed (*condense*) movements (De Meijer, 1989; Atkinson et al., 2004), and Dael described fear motor expressions as involving backward body lean (*retreat*) (Dael et al., 2012b).

Feeling of happiness was predicted by *jumping* and *rhythmic movements*, which are a fundamental part of many folk dances all over the world. People often dance in order to elevate their mood (e.g., when going to dancing clubs), and these motor characteristics of the dance movements can explain the mechanism behind this cross-cultural effect. Happiness was also predicted to be enhanced by *lightness* and *free flow*. In order for a movement to be light and free, one has to generate the minimal amount of force necessary for achieving the required limb displacement. When we are stressed, our muscles become tense as part of getting ready to fight or flight, there is increased co-contraction, and each movement requires more muscle activation in order to overcome this co-contraction. The feeling of happiness produced by free and light movements might be the result of the proprioceptive feedback from the muscles to the brain, which similar to what happens during relaxation, signals to the brain that the muscles are minimally activated, i.e., we are not in a stressful situation. Additional motor elements that predicted feeling happy were enlarging the shape of the body in the horizontal (*spread*) and vertical (*rise*) direction as well as *upward* movements in space. Moving with these motor elements causes our body to become bigger and larger, and produces a feeling of dominance and power (Carney et al., 2010). Such feelings of being powerful produce a sensation of security and reduce stress, which, again, might be the reason why we feel happy when performing such movements.

Similar to anger and fear, the motor elements that we found as predicted happiness characterize movements that have been used to induce happiness. In a previous study that demonstrated happiness enhancement through posture, subjects were asked to sit as straight as they can, which means that they had to *rise* their torso (Flack et al., 1999). In another study, happiness was not measured directly, but dancing a dance (*rhythmic movements*) that incorporated small *jumps* decreased depression and increased vitality (Koch et al., 2007). The motor elements that we found enhanced happiness characterize also some of the motor expressions of happiness described in other studies. These included repetitive *(rhythmic*), vertical (*upward*) movements of the arms (Dael et al., 2012a), and loose (*free flow*) (Montepare et al., 1999), *light* (Lourens et al., 2010), and expanded (*spread*) movements (Montepare et al., 1999; Crane and Gross, 2013).

Feeling sad was predicted by movements that were done with *passive weight, sinking, head down, and arm(s) to upper body*. Passive weight, sinking and head down characterize movements performed with minimal energy expenditure and communicate submission. The association between sadness and this type of movements is in accordance with evolutionary theories of sadness, which postulate that we are sad when we encounter adversities, which require us to save our energy and avoid confrontations with stronger animals, until we can regroup and regain our strength back (Hagen, 2011). Moving with minimal energy expenditure enables to use the little energy we have in such circumstances to overcome the adversity, and so does signaling of submissiveness through movements, which is done in order to avoid confrontation with other animals and to be left alone to recuperate. Bringing arms to the upper body may be done to either hug oneself, to touch one's face, or to use the hands as a rest for the heavy head. Using the arms as a rest for the head is congruent with the feeling of lack of energy that characterizes sadness. Hugging oneself brings comfort, and so does touching one's face, which is one of the displacement activities that serve in human and non-human primates both as an indicator to stress and as an adaptive response that reduces stress (Troisi, 2002). The association between these two behaviors and sadness is congruent with the theory that sadness has evolved as a reaction to separation from the mother and serves to re-establish physical proximity (Hagen, 2011).

In previous studies that have elicited sadness through posture, subjects were asked, among other motor behaviors to drop their *head down*, to droop their shoulders and let their rib cage fall (*sinking*) and to let the rest of their body go limp (passive weight) (Duclos et al., 1989; Flack et al., 1999; Duclos and Laird, 2001). Studies that described whole body sad expressions portrayed those as contracted, shrinking posture (Montepare et al., 1999; Gross et al., 2012; Crane and Gross, 2013) which is equivalent to *sinking*, with loss of muscle tone (Dael et al., 2012a) and collapsed or slumped torso (*heavy/passive weight)* (Wallbott, 1998; Michalak et al., 2009), and with the *head down* (Wallbott, 1998; Michalak et al., 2009; Roether et al., 2009).

Although for each emotion we found several motor elements whose existence predicted the elicitation of that emotion through movement, Table 4 shows that it was not necessary for all the predictors of a certain emotion to appear in a motor sequence (motif) in order for that motor sequence to generate or enhance that emotion. Some motifs caused the people who performed them to feel the emotion associated with them even when they included only one of the motor elements predicting that emotion, such as in the case of the “happy” motif 302.001, or only two predictors motor elements, as in the case of the “sad” motif 29.013, the “angry” motif 202.001, or the “fear” motif 48.02. Moreover, Table 4 demonstrates the importance and effect of the “strength” of a predictor as it is expressed through (1) the size of its estimate in the logistic regression, and (2) the amount of that motor element in the motor sequence (the score of that element in the motif): The “happy” motif 302.001, for example, included one predictor for happiness: Body-Action:Jump, and two predictors for anger: Shape-Sagital:Advance, and Effort-Time:Sudden. In spite of including two predictors for anger, this motif was experienced as generating happiness and had the highest “%correct^*^intensity felt” value for happiness, probably because the happiness predictor Body-Action:Jump had a larger estimate (1.5676) than the estimates of the anger predictors Shape-Sagital:Advance (0.7609) and Effort-Time:Sudden (0.8119), and because the motif included more of it (a score of 2 compared to a score of 1 for Shape-Sagital:Advance and for Effort-Time:Sudden).

These descriptive findings are important as they can facilitate personalizing motor interventions for emotion regulation. For example, happiness could be enhanced by choosing to incorporate into one's daily movements only those motor characteristics that feel comfortable and natural to adopt. Using only some of the motor elements that enhance a certain emotion, instead of all of them, could be made up for, by using “stronger” predictors for a longer duration or more frequently. In addition, the effects of some of these motor elements could be activated by one getting involved and becoming active in other movement disciplines that incorporate these motor elements. For example: in Alexander Technique people learn to effortlessly elongate (*rise* with *light weight*) their body, and in yoga there is constant emphasis on opening the chest (*spreading*). The findings of the negative predictors are also important for personalization of emotion-regulation intervention. Diminishing the time spent moving with negative predictors could be an important strategy to reduce some of the negative emotions and should be emphasized in people who tend to incorporate those motor elements a lot into their daily movements.

Our findings regarding the motor elements associated with specific emotions have numerous potential applications. One important application, as already suggested, is using this knowledge for emotion regulation. Knowing these predictors will provide people a tool to help regulate their emotions through their motor behavior, by incorporating into their daily movements those motor elements that enhance happiness, and by avoiding or decreasing motor behaviors that include elements that enhance negative emotions. People could be taught to identify those motor characteristics in their movements by health-related or movement professionals, who will be trained by LMA experts how to teach this material to patients. Moreover, we have recently started to develop an automated Laban-motor-elements recognition, using the inexpensive Kinect camera (Bernstein et al., 2015a,b). Such camera could be placed in a patient's home, capture his everyday movements, and give feedback as to which motor elements he is using in his movements. This feedback can guide patients how to change their movements in order to affect their emotion in the desired direction. In addition, although happiness is generally a goal, some people may need to allow themselves to experience anger, sadness or fear. Dance therapists could help such patients to experience these emotions by directing them how to move, using our findings. Additional application to the knowledge of the associations between certain emotions and specific motor elements is teaching actors, politicians or public speakers how to convey through their body language the emotional message they want to communicate. Similarly, by adding certain motor elements to movements, one can add emotion to a neutral movement and create bodily emotional expressions in robots, animations, avatars, or virtual reality characters to make them move and behave more human-like (Masuda and Kato, 2010). Such application will significantly advance the field of human-agent interaction, as users will be able to perceive robots' emotions and form attachment to the robot more easily, if the robot can express emotions naturally.

Our study has several limitations: First, the motor elements that were tested in this study were chosen based on their appearance in the clips produced by Atkinson et al. (2004, 2007). The movements in those clips were performed by a small set of professional actors who exaggerated their emotional expressions. It is possible that if we would have extracted the motor elements from spontaneous movements of people expressing their feelings during natural situations and in a variety of cultural settings, we would have come up with a different set of elements to be tested in this study, and consequently with a different list of predictors. Thus, it is possible that the list of predictor motor elements that we have identified is not complete. Moreover, the participants in the study who rated their feelings following the movements were all people familiar with LMA, and it is possible that people who are not trained in movement would have different associations between movements and emotions and a different reaction to movements that include those motor elements. We could not have performed this study with people who are not trained in LMA as they wouldn't know to read the motifs, and because it takes time and practice to learn how to produce the motor quality represented by each motor element. Nevertheless, this limitation will be overcome in two follow-up studies whose purpose is to verify and strengthen the associations found in this study between specific motor elements and certain emotions, and their relevance and existence also among regular/novice people: in the first we will investigate emotion recognition by regular/novice people from video clips showing movements incorporating various combinations of the motor elements that were found as predictors of each emotion. In the second follow-up study we will induce different emotions in regular/novice people and will assess whether the predictor motor elements that were found in this study are present in the motor expressions of the associated induced feelings. Another limitation is that although the motor elements found as predictors made sense based on evolutionary theories and therefore suggest that they are universally valid, it is possible that cultural differences affect these predictors. Our study included participants from Europe, Australia, South America and North America, but lack participants from Asia and Africa, and we did not analyze our data to see if there were differences between geographic locations due to insufficient numbers from each location for statistical analysis. Lastly, previous reading and moving of motifs might have influenced successive reading. To overcome this limitation we presented the motifs to the participants in random order.

## Conclusion

In conclusion, our study has identified sets of motor characteristics that predict the elicitation or enhancement of each of the emotions: anger, fear, happiness, and sadness, when moving those characteristics. Knowing these predictors will provide people a tool to help regulate their emotions through their motor behavior, by incorporating into their daily movements those motor elements that enhance happiness, and by avoiding or decreasing motor behaviors that include elements that enhance negative emotions. Moreover, using motor elements to enhance specific emotions, as opposed to using specific movements, will enable to personalize this process of emotion regulation through movement. Additional applications in the fields of human-robot interaction and emotional communication also exist. In sum, our findings have not only theoretical value but a practical importance as well.

## Author contributions

TS conceived the study. She contributed to the design of the study, to data acquisition and results interpretation, and she wrote the manuscript. RT contributed to the design of the study, to data acquisition and to results interpretation. She revised the manuscript. KW contributed to the design of the study. She performed the statistical analysis, and revised the manuscript. All authors approve the manuscript and agree to be accountable for all aspects of the work.

## Funding

The Research Open Access Article Publishing (ROAAP) Fund of the University of Illinois at Chicago, provided financial support toward the open access publication fee for this article.

### Conflict of interest statement

The authors declare that the research was conducted in the absence of any commercial or financial relationships that could be construed as a potential conflict of interest.
